# Rituximab Therapy for Primary Membranous Nephropathy in a Chinese Cohort

**DOI:** 10.3389/fmed.2021.663680

**Published:** 2021-05-20

**Authors:** Shuang Gao, Zhao Cui, Xin Wang, Yi-miao Zhang, Fang Wang, Xu-yang Cheng, Li-qiang Meng, Fu-de Zhou, Gang Liu, Ming-hui Zhao

**Affiliations:** ^1^Renal Division, Peking University First Hospital, Beijing, China; ^2^Institute of Nephrology, Peking University, Beijing, China; ^3^Key Laboratory of Renal Disease, Ministry of Health of China, Beijing, China; ^4^Key Laboratory of Chronic Kidney Disease (CKD) Prevention and Treatment, Ministry of Education of China, Beijing, China; ^5^Peking-Tsinghua Center for Life Sciences, Beijing, China

**Keywords:** primary membranous nephropathy, CD20 monoclonal antibody, rituximab, anti-PLA2R antibody, first line treatment

## Abstract

**Background:** Rituximab has become one of the first-line therapies for the treatment of moderate and high-risk primary membranous nephropathy (pMN). We retrospectively reviewed 95 patients with pMN who received rituximab therapy and focused on the therapeutic effects and safety of this therapy in a Chinese cohort.

**Methods:** Ninety-five consecutive patients with pMN diagnosed by kidney biopsy received rituximab and were followed up for >6 months. Four weekly doses of rituximab (375 mg/m^2^) was adopted as the initial administration. Repeated single infusions were administrated to maintain B cell depletion levels of <5 cells/mL.

**Results:** A total of 91 patients completed rituximab therapy with the total dose of 2.4 (2.0, 3.0) g; 64/78 (82.1%) patients achieved anti-PLA2R antibody depletion in 6.0 (1.0, 12.0) months; 53/91 (58.2%) patients achieved clinical remission in 12.0 (6.0, 24.0) months, including complete remission in 18.7% of patients and partial remission in 39.6% of patients. Multivariate logistic regression analysis showed that severe proteinuria (OR = 1.22, *P* = 0.006) and the persistent positivity of anti-PLA2R antibodies (OR = 9.00, *P* = 0.002) were independent risk factors for no-remission. The remission rate of rituximab as an initial therapy was higher than rituximab as an alternative therapy (73.1 vs. 52.3%, *P* = 0.038). Lastly, 45 adverse events occurred in 37 patients, but only one patient withdrew from treatment due to severe pulmonary infection.

**Conclusion:** Rituximab is a safe and effective treatment option for Chinese patients with pMN, especially as an initial therapy.

## Introduction

Primary membranous nephropathy (pMN) is an autoimmune kidney disease and the most common cause of adult nephrotic syndrome (1, 2). The M-type phospholipase A2 receptor (PLA2R) expressed on podocytes is the most representative *in situ* antigen, and anti-PLA2R IgG4 antibodies are detected in nearly 70% of patients (2, 3), which bind to the antigen and form a subepithelial immune complex.

Nearly 30% of patients with pMN are able to achieve spontaneous remission; however, remission is less likely to occur in moderate and high-risk patients, namely patients with massive proteinuria, with or without normal and stable kidney function (4, 5). For those patients, cyclophosphamide combined with corticosteroids, or calcineurin inhibitors (cyclosporine or tacrolimus) have been widely administrated (1). With the advances in our understanding of the pathogenesis of pMN, there has been increased interest in rituximab, a monoclonal antibody against the CD20 antigen found on the surface of B cells. Remuzzi et al. (6) reported the first clinical application of rituximab for pMN treatment in 2002, showing the superiority of rituximab therapy to traditional immunosuppressive regimens in the short term. In 2012, Ruggenenti et al. (7) reported a 65% remission rate in 7.1 months, and a similar remission rate was observed in the GEMRITUX study. In addition, the authors of the GEMRITUX study found a higher rate of anti-PLA2R antibody depletion (56%) compared to the non-immunosuppressive treatment (4%) (8). In the MENTOR study, rituximab showed superiority in its therapeutic effect compared to cyclosporine (9). In the latest version of the KDIGO clinical practice guidelines (2020) on glomerular diseases, rituximab has been listed as a first-line therapy for moderate and high-risk patients with pMN (4).

Race has a certain influence on the pathogenesis and treatment of pMN. Xie et al. (10) mapped the human leukocyte antigen (HLA) locus, and reported that DRB1^*^1501 is the major risk allele in Asians, DQA1^*^0501 in Europeans, and DRB1^*^0301 in both races, suggesting that T cells may activate the PLA2R pathway on diverse epitopes. Additionally, Zhang et al. (11) proposed another mechanism of pMN pathogenesis in Chinese patients, namely environmental pollution. Long-term exposure to PM2.5 (particulate matter with diameter ≤ 2.5 microns in the atmosphere, also known as lung accessible particles) is a major risk factor for pMN, and the study by Zhang et al. showed that when the PM2.5 concentration is higher than 70 g/m^3^, the incidence rate of pMN is directly proportional to PM2.5 concentration. Therefore, it is necessary to study the effect of rituximab on Chinese patients with pMN because of its specificity shown above.

In our study, rituximab therapy was administrated to a cohort of Chinese patients with pMN as an initial or alternative therapy, between January 2013 and 2020. To our knowledge, this is the largest-scale retrospective study of rituximab treatment in Asian patients with pMN.

## Methods

### Participants

A total of 95 consecutive patients treated at Peking University First Hospital from January 2013 to 2020 were retrospectively reviewed and fulfilled the following criteria: (i) biopsy-proven pMN; (ii) received rituximab as an initial or alternative therapy; and (iii) were not accompanied by chronic infectious diseases that affect immunosuppressive therapy, such as tuberculosis and acquired immune deficiency syndrome (AIDS). Clinical data were collected from the patient's medical records.

### Rituximab Treatment and Follow-Up

Four weekly doses of rituximab therapy (375 mg/m^2^) was adopted as the initial administration. The clinicians adjusted the dosage and/or frequency based on the individual characteristics of the patients, such as kidney function. Rituximab was infused as previously described (10). CD19+ B lymphocyte depletion was defined as <5 cells/mL and was evaluated at each follow-up. After the initial administration, repeated infusions of rituximab were administrated at 375 mg/m^2^ for single usage, once B cell levels reached >5 cells/mL within a few days.

All the patients underwent a series of follow-up appointments after initial rituximab administration at month 0, 1, 3, 6, and at subsequent 6-month intervals until the endpoint. The endpoint was end-stage renal disease (ESRD) or death. Laboratory evaluations, including 24-h proteinuria, serum albumin, creatinine, eGFR, anti-PLA2R antibodies, and circulating B cell amount, were performed at baseline and at every visit. Adverse events related to rituximab were evaluated during drug infusion and the entire follow-up period.

### Treatment Responses and Renal Outcomes

To evaluate therapeutic responses, complete remission was defined as proteinuria <0.3 g/24 h, and partial remission was defined as proteinuria <3.5/24 h or a reduction of >50% from baseline, with improvement or normalization of serum albumin concentration, and stable or elevated <30% from baseline of serum creatinine. Patients who did not reach remission were considered non-responders. The recurrence of proteinuria >3.5 g/24 h after a period of remission was regarded as relapse (9, 12). To evaluate kidney outcomes, the primary endpoint was ESRD with eGFR <15 mL/min/1.73 m^2^ or receiving dialysis, and the secondary endpoint was the elevation of serum creatinine or a >50% reduction of eGFR from baseline (12).

### Statistical Analysis

Statistical analysis was performed using Statistical Product and Service Solutions (SPSS) 23.0 (IBM, New York, USA). Data following a non-normal distribution were presented as median [interquartile range (IQR)]. For Data following a normal distribution, quantitative and semi-quantitative data were expressed as mean ± standard deviation (SD). A *t*-test was used to assess differences between quantitative data and Kruskal–Wallis test or Mann–Whitney *U*-test was used for semi-quantitative data. Qualitative data were expressed as amount (percentage) and a chi-squared test or one-way variation analysis (ANOVA) were used to assess the differences. All probabilities were two-sided and a *p* < 0.05 was considered statistically significant. Logistic regression and Cox regression analyses were performed to confirm potential risk or protection factors of treatment responses.

## Results

### Patients

**Figure 1:**
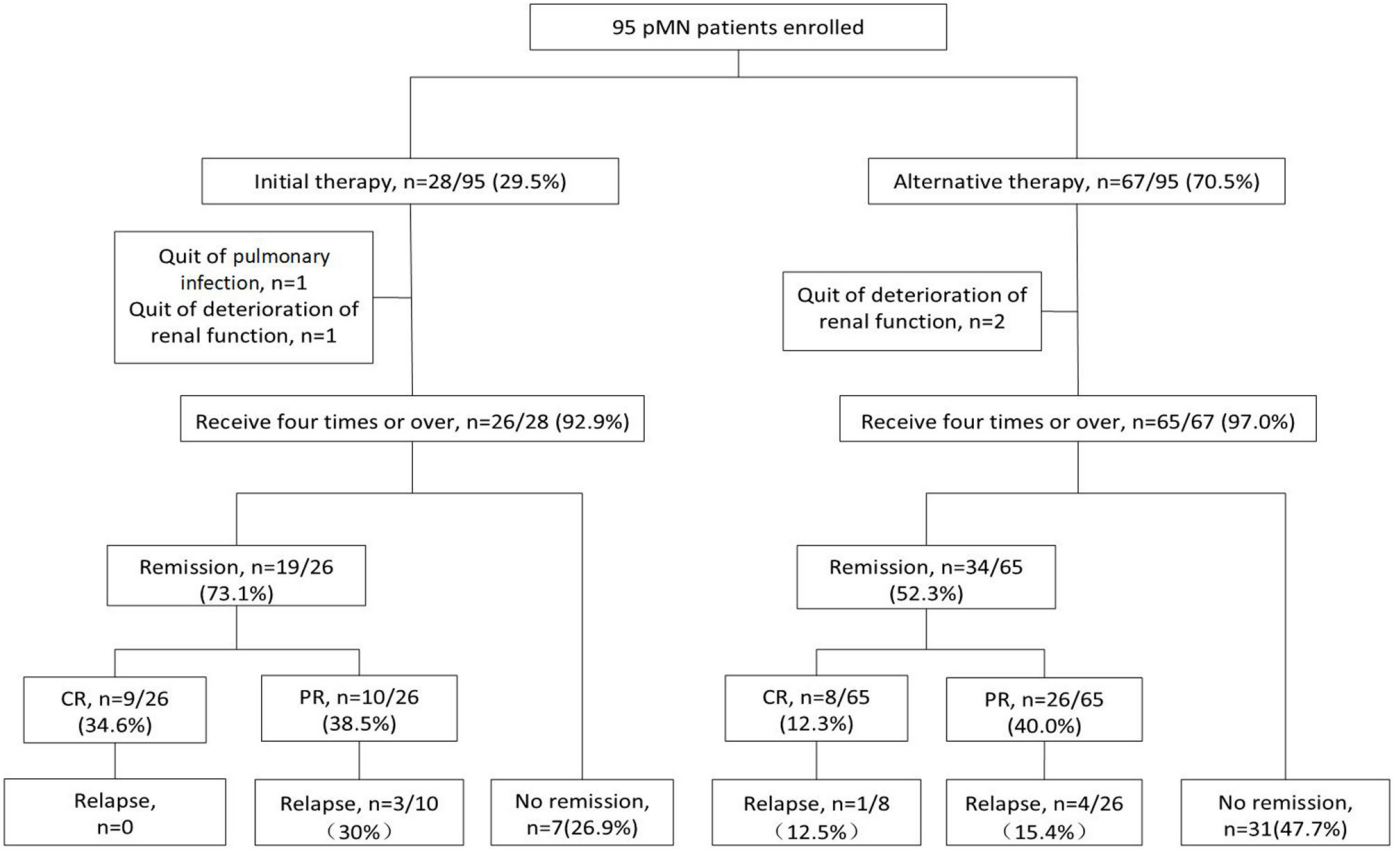
Flow chart of the patients with pMN receiving rituximab therapy. There were 95 PMN patients enrolled, with 28 patients received rituximab as initial therapy and 67 as alternative therapy. There were 91 (95.8%) patients who accomplish the first administration of four times weekly usage and the remaining 4 (4.21%) patients did not. One patient discontinued treatment due to severe pulmonary infection, and three patients progressed into ESRD before the fourth infusion of rituximab. The remission rate was 73.1% (34.6% CR and 38.5% PR) in initial group and 52.3% (12.3% CR and 40.0% PR) in alternative group. 3/10 (30%) PR patients relapsed in initial group. 1/8 (12.5%) CR patients and 4/26 (15.4%) PR patients relapsed in alternative group. CR, complete remission; PR, partial remission.

There were 95 patients with pMN who received rituximab treatment as an initial or alternative therapy (Figure 1). The clinical and pathological characteristics of the patients at baseline is presented in Table 1. There were 83 male patients and 12 female patients, with a median age of 47 (30, 59) years old. The level of proteinuria was 9.6 (5.7, 13.4) g/24 h, serum albumin was 21.7 (18.9, 28.1) g/L, and eGFR was 63.8 (39.4, 93.9) mL/min/1.73 m^2^. There were 82 (86.3%) patients positive for anti-PLA2R antibodies (>20 U/mL), with a median level of 122.5 (47.0, 309.0) U/mL. All patients underwent kidney biopsy and were diagnosed with pMN. Granular deposits of IgG were observed in all patients, with a median staining intensity of 3+, and the staining intensities of IgG1, IgG2, IgG3, and IgG4 were 2+, 1+, 1+, and 3+, respectively. Additionally, 93.5% of the patients had stage I or stage II membranous injury, 6.5% had stage III, and no patient had stage IV.

**Table 1:** Baseline characteristics of patients with pMN included in this study.

Parameters^*^	**Total (*n* = 95)**	**Initial therapy (*n* = 28)**	**Alternative therapy (*n* = 67)**	***P*^§^**
Gender [male, *n* (%)]	83 (86.5%)	22 (75.0%)	61 (91.0%)	0.057
Age (years)	47.0 (30.0, 59.0)	51.0 (32.3, 67.5)	45.0 (28.0, 58.0)	**0.020**
Proteinuria (g/24 h)	9.6 (5.7, 13.4)	7.3 (4.6, 12.2)	10.6 (6.3, 15.0)	**<0.001**
Serum albumin (g/L)	21.7 (18.9, 28.1)	23.2 (19.8, 30.1)	20.8 (17.8, 28.1)	**0.006**
Anti-PLA2R antibody positivity, *n* (%)	82 (86.3%)	27 (96.4%)	55 (82.1%)	0.427
Anti-PLA2R antibodies (U/mL)	122.5 (47.0, 309.0)	243.0 (73.0, 460.0)	94.0 (39.0, 213.0)	**0.003**
Serum creatinine (μmol/L)	110.7 (85.3, 161.0)	88.1 (64.6, 127.4)	131.6 (92.5, 180.0)	0.078
eGFR^†^ (mL/min/1.73 m^2^)	63.8 (39.4, 93.9)	84.8 (53.5, 108.5)	52.6 (37.6, 87.2)	0.066
**Pathological features**
PLA2R staining (0–4+)	1.8 (1.0, 2.0)	1.3 (1.0, 2.0)	2.0 (1.0, 2.0)	0.507
IgG deposit (0–4+)	3.0 (3.0, 4.0)	3.3 (2.5, 4.0)	3.0 (3.0, 3.0)	0.290
Stages of membranous injury	2.0 (1.0, 2.0)	2.0 (1.0, 2.0)	2.0 (1.0, 2.0)	0.807

* *Median (IQR)*.

§ *Comparisons between initial therapy group and alternative therapy group*.

† *eGFR, estimated glomerular filtration rate. The bold values represents P <0.05*.

A total of 28 patients received rituximab as initial therapy and 67 as alternative therapy (Figure 1). Following diagnosis by percutaneous renal biopsy, the alternative therapy group was administered 2 (1–6) courses of immunosuppressant therapy before rituximab treatment, including cyclosporine combined with steroids in 53 (76.8%) patients, cyclophosphamide combined with steroids in 43 (62.3%) patients, tacrolimus in 36 (52.2%) patients, mycophenolate mofetil in 23 (33.3%) patients, and leflunomide in 6 (8.7%) patients. Among the 67 patients in the alternative therapy group, 35 (52.2%) achieved remission and the remaining 32 (47.8%) had no response.

In general, the alternative therapy group was younger and presented with more severe condition, both of which predicted worse responses (Table 1). The initial therapy group were older [51.0 (32.3, 67.5) vs. 45.0 (28.0, 58.0) years, *p* = 0.020], and presented with lower levels of proteinuria [7.3 (4.6, 12.2) vs. 10.6 (6.3, 15.0) g/24 h, *p* < 0.001], higher levels of serum albumin [23.2 (19.8, 30.1) vs. 20.8 (17.8, 28.1), *p* = 0.006], and higher levels of anti-PLA2R antibodies [243.0 (73.0, 460.0) vs. 94.0 (39.0, 213.0), *p* = 0.003], compared to the alternative therapy group. Gender, anti-PLA2R antibody positivity, serum creatinine, eGFR, and pathological features were comparable between the two groups (*p* > 0.05).

### Rituximab Dosage

There were 91 (95.8%) patients who received four or more rituximab infusions, with a total dose of 2.4 (2.0, 3.0) g. The remaining 4 (4.21%) patients did not complete the first administration of four doses (Figure 1). One patient discontinued treatment due to severe pulmonary infection, and three patients progressed into ESRD before the fourth infusion of rituximab. Compared to the initial therapy group, the alternative therapy group required more rituximab infusions (4.9 ± 1.4 vs. 4.2 ± 0.6, *P* = 0.021), and achieved a lower remission rate (52.3 vs. 73.1%, *P* = 0.038; Table 2).

**Table 2:** Details of rituximab therapy and follow-up.

**Parameters**	**Total (*n* = 95)**	**Initial therapy (*n* = 28)**	**Alternative therapy (*n* = 67)**	***P***
Rituximab treatments^†^	*n* = 91	*n* = 26	*n* = 65	
Total dose (g)	2.4 (2.0, 3.0)	2.4 (2.4, 2.8)	2.4 (2.0, 3.0)	0.604
Infusion times (mean ± SD)	4.7 ± 1.3	4.2 ± 0.6	4.9 ± 1.4	**0.021**
**Treatment responses**
Remission, *n* (%)	53 (58.2%)	19 (73.1%)	34 (52.3%)	**0.038**
CR/PR	17/36	9/10	8/26	0.173
Relapse, *n* (%)	8/53 (15.1%)	3/19 (15.8%)	5/34 (14.7%)	0.577
No response, *n* (%)	38 (41.8%)	7 (26.9%)	31 (47.7%)	**0.038**
Follow-up time (months)	24.0 (7.5, 36.0)	18.0 (6.0, 24.0)	24.0 (12.0, 36.0)	0.065

† *91 patients who received rituximab for four times or over. The bold values represents P <0.05*.

### Treatment Responses

#### Immunological Responses

In the 91 patients who received at least four infusions of rituximab, 78 (85.7%) patients had positive anti-PLA2R antibodies at baseline, with a median level of 120.2 (45.1, 306.4) U/mL. After rituximab therapy, 64/78 (82.1%) patients achieved immunological remission with anti-PLA2R antibody depletion (<20 U/mL) in 6.0 (1.0, 12.0) months. Among the antibody-deleption group, 48/64 (75.0%) patients achieved clinical remission, with 17 achieving complete remission and 31 achieving partial remission. There were 14 (17.9%) patients who maintained positive anti-PLA2R antibodies during the entire follow-up period, although their B cell levels were maintained at <5/mL. Among these 14 patients, only 2 (14.3%) patients achieved clinical remission. There was a significant difference in remission rate between antibody-deleption and antibody-undeleption group (*P* < 0.001). During follow-up, antibodies reoccurred in 12/64 (18.8%) patients, in which 9/12 (75.0%) patients were non-responders, 2 (16.7%) patients relapsed, and 1 (8.3%) patient remained in partial remission.

#### Clinical Responses

**Figure 2:**
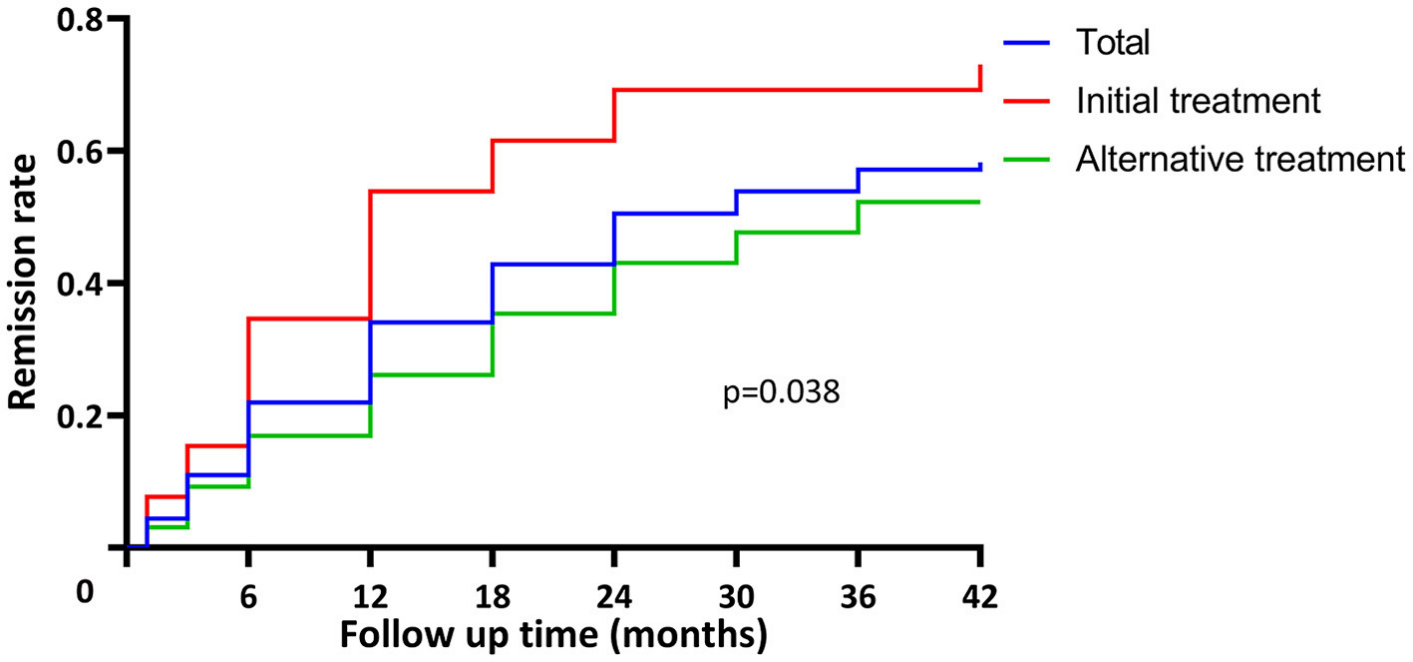
Time to remission in patients with pMN receiving rituximab treatment. The remission rate was 58.2% (53/91) in total. The initial therapy group had higher remission rate compared to the alternative therapy group [19/26 (73.1%) vs. 34/65 (52.3%), *P* = 0.038] and achieved remission sooner [12.0 (6.0, 18.0) vs. 15.0 (6.0, 24.0) months].

During the follow-up period of 24.0 (7.5, 36.0) months, clinical remission was achieved in 53/91 (58.2%) patients at 12.0 (6.0, 24.0) months, including 17 (18.7%) with complete remission and 36 (39.6%) with partial remission. Anti-PLA2R antibodies (*P* = 0.037) and proteinuria (*P* < 0.001) decreased in all patients receiving rituximab therapy, especially in responders. eGFR remained stable in responders and decreased significantly in non-responders (*P* < 0.001). The initial therapy group had a higher remission rate compared to the alternative therapy group [19/26 (73.1%) vs. 34/65 (52.3%), *P* = 0.038] and achieved remission sooner [12.0 (6.0, 18.0) vs. 15.0 (6.0, 24.0) months] (Figure 2).

For the initial therapy group, the persistent positivity of anti-PLA2R antibodies (OR = 45.00, 95% CI = 3.35–603.99, and *P* = 0.004) was the only risk factor to treatment failure. For the alternative therapy group, univariate logistic regression analysis showed that higher levels of proteinuria (OR = 1.18, 95% CI = 1.06–1.31, and *P* = 0.002), higher levels of anti-PLA2R antibodies (OR = 1.01, 95% CI = 1.00–1.01, and *P* = 0.024), and the persistent positivity of anti-PLA2R antibody (OR = 11.02, 95% CI = 2.78–43.75, and *P* = 0.001) were risk factors to no remission. In contrast, older age (OR = 0.96, 95% CI = 0.93–0.99, and *P* = 0.016) and a higher concentration of serum albumin (OR = 0.89, 95% CI = 0.82–0.97, and *P* = 0.008) were protective factors. Multivariate logistic regression analysis showed that a higher level of proteinuria (OR = 1.19, 95% CI = 1.01–1.39, and *P* = 0.033) was the independent risk factor to no remission after rituximab treatment (Table 3).

**Table 3:** The risk factors for no-remission of patients with pMN receiving rituximab for initial therapy or alternative therapy (logistic regression).

	**Univariate analysis**		**Multivariate analysis**	
	**OR (95% CI)**	***P-*value**	**OR (95% CI)**	***P-*value**
**Initial therapy (*****n*** **=** **26)**				
Gender (male)	6.65 × 10^8^ (0.00, ∞)	0.999		
Age (years)	1.01 (0.97, 1.06)	0.598		
Urinary protein (g/24 h)	1.08 (0.90, 1.30)	0.424		
Serum albumin (g/L)	1.00 (0.90, 1.10)	0.963		
Anti-PLA2R antibodies (U/mL)	1.00 (0.99, 1.01)	0.380		
Persistent positivity of antibody	45.00 (3.35, 603.99)	**0.004**		
eGFR (mL/min/1.73 m^2^)	0.97 (0.95, 1.00)	0.086		
Total dose of rituximab	1.00 (0.99, 1.01)	0.785		
Infusion times of rituximab	1.24 (0.30, 5.15)	0.769		
**Alternative therapy (*****n*** **=** **65)**				
Gender (male)	2.07 (0.35, 12.13)	0.421		
Age (years)	0.96 (0.93, 0.99)	**0.016**	0.96 (0.91, 1.01)	0.115
Round of previous immunotherapy	1.01 (0.66, 1.55)	0.964		
Urinary protein (g/24h)	1.18 (1.06, 1.31)	**0.002**	1.19 (1.01, 1.39)	**0.033**
Serum albumin (g/L)	0.89 (0.82, 0.97)	**0.008**	0.99 (0.87, 1.13)	0.881
Anti-PLA2R antibodies (U/mL)	1.01 (1.00, 1.01)	**0.024**	1.01 (1.00, 1.01)	0.052
Persistent positivity of antibody	11.02 (2.78, 43.75)	**0.001**	5.59 (0.96, 32.46)	0.055
eGFR (mL/min/1.73 m^2^)	1.00 (0.98, 1.01)	0.514		
Total dose of rituximab	1.00 (1.00, 1.00)	0.732		
Infusion times of rituximab	1.09 (0.77, 1.55)	0.616		

*The bold values represents P <0.05*.

**Figure 3:**
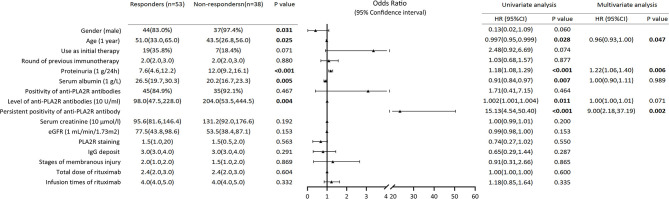
Composite comparisons of clinical features of patients with pMN between responders and non-responders. Compared to the responders, the non-responders were more likely to be young man with higher levels of urinary protein, lower concentrations of serum albumin and higher levels of anti-PLA2R antibodies. The univariate logistic regression analysis showed that the higher level of urinary protein and the persistent positivity of anti-PLA2R antibodies were risk factors for not achieving remission after rituximab treatment. Multivariate logistic regression analysis showed that the higher level of urinary protein and the persistent positivity of anti-PLA2R antibodies were independent risk factors to rituximab treatment failure, while older age was an independent protective factor.

Compared to the responders, the non-responders were more likely to be male (97.4 vs. 83.0%, *P* = 0.031), younger [43.5 (26.8, 56.0) vs. 51.0 (33.0, 65.0), *P* = 0.025), and have higher levels of urinary protein [12.0 (9.2, 16.1) vs. 7.6 (4.6, 12.2), *P* < 0.001], lower concentrations of serum albumin [20.2 (16.7, 23.3) vs. 26.5 (19.7, 30.3), *P* = 0.005], and higher levels of anti-PLA2R antibodies [204.0 (53.5, 444.5) vs. 98.0 (47.5, 228.0), *P* = 0.004] at baseline. The univariate logistic regression analysis showed that the higher level of urinary protein (OR 1.18, 95% CI 1.08–1.29, *P* < 0.001), and the persistent positivity of anti-PLA2R antibodies (OR = 15.13, 95% CI = 4.54–50.40, and *P* < 0.001) were risk factors for no remission after rituximab treatment. In contrast, older age (OR = 1.00, 95% CI = 0.95–1.00, and *P* = 0.028) and a higher concentration of serum albumin (OR = 0.91, 95% CI = 0.84–0.97, and *P* = 0.007) were protective factors. Multivariate logistic regression analysis showed that the higher level of urinary protein (OR = 1.22, 95% CI = 1.06–1.40, and *P* = 0.006) and the persistent positivity of anti-PLA2R antibodies (OR = 9.00, 95% CI = 2.18–37.19, and *P* = 0.002) were independent risk factors to rituximab treatment failure, while older age (OR = 0.96, 95% CI = 0.93–1.00, and *P* = 0.047) was an independent protective factor (Figure 3).

During the follow-up period, 8/53 (15.1%) patients relapsed after clinical remission (one complete remission and seven partial remission). Among them, three patients were from the initial therapy group and five from the alternative therapy group. After relapsing, one patient achieved partial remission after receiving rituximab therapy again, and the remaining seven patients did not achieve remission in the follow-up period after receiving cyclophosphamide and/or calcineurin inhibitor therapy. Compared to the patients who stayed in remission, relapsed patients presented with lower eGFR [40.1 (19.2, 79.0) vs. 79.5 (46.8, 102.5) mL/min/1.73 m^2^, *P* = 0.034] at baseline. Univariate logistic regression analysis showed that higher eGFR (OR = 0.97, 95% CI = 0.95–1.00, *P* = 0.045) was the only protective factor to relapse (Table 4).

**Table 4:** The risk factors for relapse of patients with pMN receiving rituximab.

	**Univariate analysis**	
	**OR (95% CI)**	***P*-value**
Gender	1.81 (0.30, 10.86)	0.517
Age (years)	1.05 (1.00, 1.11)	0.064
Initial therapy	0.92 (0.19, 4.36)	0.916
Round of previous immunotherapy	1.85 (0.77, 4.48)	0.172
Urinary protein (g/24 h)	1.07 (0.92, 1.24)	0.368
Serum albumin (g/L)	0.93 (0.82, 1.05)	0.240
Higher level of anti-PLA2R antibodies (U/ml)	1.00 (1.00, 1.01)	0.340
Positive antibody at remission	1.44 (0.14, 14.98)	0.760
eGFR (mL/min/1.73 m^2^)	0.97 (0.95, 1.00)	**0.045**
Total dose of rituximab	1.00 (0.99, 1.01)	0.744
Infusion times of rituximab	1.03 (0.46, 2.31)	0.943

*The bold values represents P <0.05*.

### Kidney Outcomes

During the follow-up period, the median eGFR of all patients decreased 5.6 (−2.9, 14.5) mL/min/1.73 m^2^, from the baseline level of 63.9 (39.6, 95.1), to an endpoint of 57.6 (30.0, 86.6) mL/min/1.73 m^2^. In the responders, eGFR remained stable compared to the baseline level (*P* = 0.750). In the non-responders, eGFR fell significantly from 53.5 (38.4, 87.1) to 41.5 (17.2, 73.4) mL/min/1.73 m^2^ (*P* = 0.038), and six patients progressed to ESRD.

There were 15/91 (16.5%) patients whose eGFR decreased more than 50% from the baseline or progressed to ESRD. Univariate Cox regression analysis indicated older age (OR = 1.03, 95% CI = 1.00–1.07, and *P* = 0.040) to be risk factor and higher eGFR (OR = 0.97, 95% CI = 0.95–1.00, and *P* = 0.020) to be a protective factor to kidney dysfunction outcome (Table 5).

**Table 5:** The risk factors for kidney dysfunction of patients with pMN receiving rituximab as eGFR decreased >50% or ESRD (Cox regression).

	**Univariate analysis**	
	**OR (95% CI)**	***P*-value**
Age (years)	1.03 (1.00, 1.07)	**0.040**
Initial therapy	0.48 (0.12, 1.88)	0.319
Round of previous immunotherapy	0.50 (0.17, 1.51)	0.217
Urinary protein (g/24 h)	1.04 (0.94, 1.16)	0.459
Serum albumin (g/L)	1.04 (0.81, 1.34)	0.734
Higher level of anti-PLA2R antibodies (U/mLs)	1.00 (0.99, 1.01)	0.487
Persistent positivity of antibody	0.94 (0.12, 7.53)	0.952
eGFR (mL/min/1.73 m^2^)	0.97 (0.95, 1.00)	**0.020**
Total dose of rituximab	1.00 (0.99, 1.01)	0.910
Infusion times of rituximab	0.88 (0.48, 1.58)	0.654
No remission	0.88 (0.23, 3.31)	0.849
Relapse	51.92 (0.00, 6.36 × 10^6^)	0.144

*The bold values represents P <0.05*.

### Safety and Adverse Effects

Among the 95 patients, 37 (38.9%) patients experienced adverse effects during rituximab treatment (Table 6). One patient withdrew from treatment due to severe pulmonary infection, and five patients (two responders and three non-responders) presented with frequent upper respiratory infections (about twice per month) in the first 6 months of rituximab administration. The most frequent, but mild, adverse effects were infusion reactions, including bronchial wheezing, rash, erythema, itching, rhinorrhea, and dysphoria. No malignancy or fatal adverse event was observed in the follow-up period. The adverse events were more frequent in non-responders than in responders (52.6 vs. 28.3%, *P* = 0.009).

**Table 6:** Adverse events in all patients with pMN receiving rituximab.

**Adverse event**	**Total**
Fatal	0
Nonfatal	45
**Myelotoxicity**
Anemia	1
Thrombocytopenia	2
**Central nervous system events**
Cerebral infarction	2
Transient loss of consciousness	1
Dizziness	5
**Respiratory system events**
Pulmonary infection	1
Frequent upper respiratory infection (URI)	5
Dyspnea	2
Cough	1
**Digestive system events**
Diarrhea	3
Abdominal pain	1
Nausea	5
Infusion reactions^†^	14
**Others**
Muscular soreness	1
Fever	1
Any events^*^
Serious adverse events (Grade ≥ 3)	3
Non-serious adverse events (Grade <3)	34
Total No. of adverse events	45
Patients with adverse events	37

† *Infusion reactions include bronchial wheezing, rash, erythema, itching, rhinorrhea, and dysphoria*.

* *The grade classification standard is WHO Toxicity Grading Scale for Determining the Severity of Adverse Events. The bold values represents P <0.05*.

## Discussion

In this study, we report the findings of rituximab therapy in a cohort of Chinese patients with pMN, and focus on the therapeutic outcomes and side effects of this treatment. We found that rituximab therapy showed good efficacy in 58.2% of all patients with pMN, with a higher rate (73.1%) of clinical remission as an initial therapy and a slightly lower rate (52.3%) as an alternative therapy. The median time to achieving remission was 12.0 months. The persistent positivity of anti-PLA2R antibodies (OR = 9.0) and more severe proteinuria (OR = 1.2) were independent risk factors to treatment failure. Side effects were observed in 38.9% of patients, consisting of mostly mild infusion reactions and several cases of respiratory infections. This retrospective analysis confirmed the therapeutic effects of rituximab therapy in Chinese patients with pMN and highlighted the necessity of antibody clearance for achieving clinical remission and better outcomes.

While the overall response rate of this cohort (58.2%) was lower than the remission rates (55–75%) from other cohorts treated with rituximab, the interval time from drug administration to clinical remission was similar between the current and previous reports (1, 5–9, 12–16). One possible reason may be the different proportions of patients enrolled in different studies. When rituximab was administrated as an initial therapy, patients achieved a higher rate (73.1%) of clinical remission, which was comparable to the remission rate of 69.1% as a first-line therapy reported by Ruggenenti et al. (7), and even better than the remission rate of 60% in the MENTOR study (9) and 64.9% in the GEMRITUX study (8). When administrated as an alternative therapy, rituximab showed a lower rate (52.3%) of clinical remission, which was comparable to the remission rate of up to 50.0% as a second-line therapy reported by Ruggenenti et al. (17). However, in the whole cohort, most (70.5%) patients received rituximab as an alternative therapy, due to the expensive cost and, thus, may explain the lower remission rate in the cohort as a whole. In general, the initial therapy group achieved a higher remission rate [19/26 (73.1%) vs. 34/65 (52.3%)] in shorter time [12.0 (6.0, 18.0) vs. 15.0 (6.0, 24.0) months] with fewer infusions (4.2 ± 0.6 vs. 4.9 ± 1.4). Thus, rituximab should be recommended for use as a first-line therapy for patients with moderate to high-risk pMN, rather than as a remedial therapy.

In this study, compared with the initial therapy group, patients in the alternative group presented with more advanced disease, which may interfere with the comparison of the therapeutic effect of rituximab. Therefore, we performed 1:1 propensity score matching to match urinary protein, albumin, renal function, anti-PLA2R antibodies, and got 17 pairs (34 patients in total). There was no significant difference in the severity of the disease between the two groups for gender, age, urinary protein, serum albumin, anti-PLA2R antibody and eGFR (*P* = 0.384, 0.096, 0.917, 0.673, 0.076, and 0.230, respectively), but there was a significant difference in the remission rate (*P* = 0.009). In the initial therapy group, 15/17 (88.2%) achieved remission, while in the alternative therapy group, only 8/17 (47.1%) achieved remission. Therefore, rituximab is more highly recommended as an initial treatment.

We previously reported the use of rituximab as an alternative therapy in our cohort (12), and most patients were enrolled in the current study, apart from a few patients who received rituximab <4 times. The remission rate increased slightly in recent years (from 41.7 to 52.3%) for several possible reasons. Firstly, all patients in the current study received the standard regimen of four infusions of rituximab (375 mg/m^2^) at the first administration and the full dose therapy may be advantageous to better treatment responses. Secondly, the longer follow-up time in the current study (24.0 vs. 12.0 months) may help identify cases of remission that occur after 12 months. It's not possible that “lack of remission” was due to inadequate follow-up time in this study, as there was no significant difference between the complete and partial remission group in follow-up time [30.0 (15.0, 36.0) vs. 24.0 (13.5, 38.3) months, *P* = 0.569].

Anti-PLA2R antibodies have been identified as a pivotal biomarker in pMN clinical practice (1, 5, 18–20), and rituximab is effective at depleting anti-PLA2R antibodies. In the current study, the level of anti-PLA2R antibodies was much higher in non-responders than in responders at both the baseline and endpoint. The MENTOR study revealed that the depletion of anti-PLA2R antibodies occurred more rapidly and at a greater magnitude and duration in the rituximab group than in the cyclosporine group (9). In this study, 82.1% of patients achieved antibody depletion in 6.0 months, and clinical remission was achieved in 12 months, confirming the efficacy of rituximab in eliminating anti-PLA2R antibodies and achieving clinical remission after antibody depletion. We further found that the persistent positivity of anti-PLA2R antibodies was one of the independent risk factors to no-remission following rituximab therapy, especially in the initial therapy group. For these patients, novel treatments that target memory B cells and long-lived plasma cells might be of consideration.

Severe proteinuria was another independent risk factor to no-remission, especially in the alternative therapy group. In this study, the baseline level of proteinuria was much higher in non-responders. The remission rate was 100% in the patients with proteinuria <4 g/24 h, 67.9% in 4–8 g/24 h, 52.4% in 8–12 g/24 h, and 42.4% in >12 g/24 h (*P* = 0.009). A pharmacokinetic experiment monitoring the serum concentration of rituximab from patients with pMN or rheumatoid arthritis showed that rituximab cleated more quickly in patients with proteinuria compared to those without proteinuria (21). However, rituximab levels did not correlate with treatment response (22, 23). Therefore, the mechanism explaining how severe proteinuria interfering rituximab treatment will require further investigation.

A significant gender bias existed in this study (12.6% female vs. 87.4% male), and may be due to a number reasons. Firstly, the incidence rate of pMN is higher in males than in females. Secondly, male patients require immunosuppressive treatment more often than female patients. Two studies by Cattran (24) reported that female patients are more likely to achieve spontaneous remission, while male patients progress more rapidly, even with comparable proteinuria (24, 25). Among the patients enrolled in this study, female patients had lower levels of proteinuria than male patients [6.19 (3.15, 8.51) vs. 10.52 (6.14, 13.60) g/24 h, *P* = 0.034] at baseline. Additionally, after rituximab treatment, female patients had a higher remission rate (90.0 vs. 54.3%, *P* = 0.031) as well. However, this result may be biased due to the small number of female patients.

Adverse events were observed in 37 (38.9%) patients, 3.2% of which were serious adverse events and 35.8% were non-serious. This was lower than previous studies that reported adverse events in 50–80% of patients and serious adverse events of 0–17% (8, 9, 12, 15). This difference might be due to the retrospective nature of this study, as some adverse events might have been forgotten, left out, or ignored from the medical records. Additionally, in the current and previous studies (9, 26), the most common adverse events were infusion-related reactions. As a biologic product, rituximab may trigger immune-mediated reactions, such as dyspnea, rash, erythema, itching, and others. However, we observed fewer infusion-related adverse events in this study compared to a previous study [14/95 (14.7%) vs. 28/100 (28.0%) (26)]. This may be due to the use of anti-allergic drugs before infusions and the restriction of infusion speed to avoid or ameliorate infusion-related events to some extent. We also found that adverse events were more frequent in non-responders than in responders. This may be due to immune-mediated sensitization syndrome that results from resistance to rituximab (1, 27). For the non-responders with immune-mediate infusion reactions, humanized anti-CD20 antibodies might be an alternative choice to avoid adverse events and drug resistance (28). Compared to steroids and cyclophosphamide therapy, rituximab was safer both in non-serious adverse events and in serious adverse events (26). Compared to cyclosporine, there was no significant difference in the incidence of adverse events from rituximab; however, fewer serious adverse events were observed in the rituximab group (9).

In conclusion, rituximab therapy was effective in the clearance of anti-PLA2R antibodies and for achieving clinical remission in a cohort of Chinse patients with pMN, especially as an initial therapy, with tolerable adverse events.

## Data Availability Statement

The raw data supporting the conclusions of this article will be made available by the authors, without undue reservation.

## Ethics Statement

The current study complies with the Declaration of Helsinki and was approved by the ethics committee of Peking University First Hospital. Written informed consent was obtained for tissue, blood, and urine samples.

## Author Contributions

ZC and M-hZ: initial idea. ZC, XW, Y-mZ, FW, X-yC, L-qM, F-dZ, GL, and M-hZ: review and comments. SG: figures. SG and ZC: concept and writing. All authors contributed to the article and approved the submitted version.

## Conflict of Interest

The authors declare that the research was conducted in the absence of any commercial or financial relationships that could be construed as a potential conflict of interest. The handling Editor declared a past co-authorship with several of the authors M-hZ, ZC.
